# A chemical genetic screen reveals that iminosugar inhibitors of plant glucosylceramide synthase inhibit root growth in Arabidopsis and cereals

**DOI:** 10.1038/s41598-018-34749-1

**Published:** 2018-11-06

**Authors:** Michael D. Rugen, Mathieu M. J. L. Vernet, Laila Hantouti, Amalia Soenens, Vasilios M. E. Andriotis, Martin Rejzek, Paul Brett, Richard J. B. H. N. van den Berg, Johannes M. F. G. Aerts, Hermen S. Overkleeft, Robert A. Field

**Affiliations:** 1 Department of Biological Chemistry, John Innes Centre, Norwich Research Park, Norwich, NR4 7UH UK; 2 Department of Metabolic Biology, John Innes Centre, Norwich Research Park, Norwich, NR4 7UH UK; 3 Leiden Institute of Chemistry, Gorlaeus Laboratories, Leiden University, Einsteinweg 55, 2300 RA Leiden, The Netherlands; 4 Department of Medical Biochemistry, Academic Medical Center, University of Amsterdam, Meibergdreef 15, 1105 AZ Amsterdam, The Netherlands; 5 Present Address: Centro de Biotecnología y Genómica de Plantas, Universidad Politécnica de Madrid-Instituto Nacional de Investigación y Tecnología Agraria y Alimentaria, Pozuelo de Alarcón, Madrid, Spain; 6 Present Address: School of Natural and Environmental Sciences, Devonshire Building, Newcastle University, Newcastle-upon-Tyne, NE1 7RU UK

## Abstract

Iminosugars are carbohydrate mimics that are useful as molecular probes to dissect metabolism in plants. To analyse the effects of iminosugar derivatives on germination and seedling growth, we screened a library of 390 *N*-substituted iminosugar analogues against Arabidopsis and the small cereal Eragrostis tef (Tef). The most potent compound identified in both systems, *N*-5-(adamantane-1-yl-ethoxy)pentyl- l-*ido*-deoxynojirimycin (l-*ido*-AEP-DNJ), inhibited root growth in agar plate assays by 92% and 96% in Arabidopsis and Tef respectively, at 10 µM concentration. Phenocopying the effect of l-*ido*-AEP-DNJ with the commercial inhibitor (PDMP) implicated glucosylceramide synthase as the target responsible for root growth inhibition. l-*ido*-AEP-DNJ was twenty-fold more potent than PDMP. Liquid chromatography-mass spectrometry (LC-MS) analysis of ceramide:glucosylceramide ratios in inhibitor-treated Arabidopsis seedlings showed a decrease in the relative quantity of the latter, confirming that glucosylceramide synthesis is perturbed in inhibitor-treated plants. Bioinformatic analysis of glucosylceramide synthase indicates gene conservation across higher plants. Previous T-DNA insertional inactivation of glucosylceramide synthase in Arabidopsis caused seedling lethality, indicating a role in growth and development. The compounds identified herein represent chemical alternatives that can overcome issues caused by genetic intervention. These inhibitors offer the potential to dissect the roles of glucosylceramides in polyploid crop species.

## Introduction

Plant carbohydrate metabolic processes, including starch, cell wall, glycolipid and glycoprotein synthesis and degradation, are essential to seedling establishment and have implications for crop quality, utilisation and pathogen resistance^1–3^. Furthermore, detailed understanding of these systems can be applied to develop better processing in value-added sectors, such as malting, brewing and food manufacture. Chemical inhibitors have proved useful as tools to gain a better understanding of how metabolic processes are controlled at the molecular level^4^. Herein we report our efforts to identify novel iminosugar inhibitors of seedling root growth and the identification of glucosylceramide synthase (GCS) as a putative target for iminosugar inhibitors in plants^5^.

**Figure 1:**
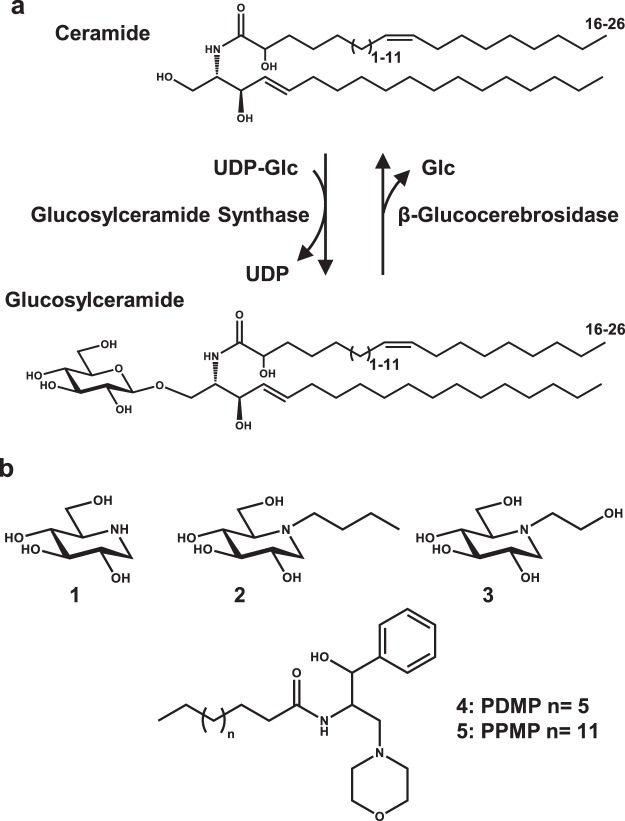
The catalytic activity and inhibitors of glucosylceramide synthase. (**a**) Glucosylceramide synthase (GCS) converts ceramide (Cer) and uridine 5′-diphosphoglucose (UDP-Glc) to glucosylceramide (GlcCer) and UDP. This represents the final step of glucosylceramide synthesis and generates the precursor for numerous complex glycosphingolipids. Plant glucosylceramides are a family of molecules which show great variety. The sphingoid base (long chain base) has three possible modifications: desaturation or hydroxylation at C4 and desaturation (cis or trans) at C8. Sphingoid bases are linked to alpha-hydroxy fatty acids with varying desaturation and chain length. β-Glucocerebrosidase (GCase) catalyses the hydrolysis of GlcCer to generate glucose (Glc) and Cer. (**b**) The structures of archetypal GCS inhibitor scaffolds: **1** 1-deoxynojirimycin (DNJ), **2** *N*-butyl-1-deoxynojirimycin (*N*-Bu-DNJ), **3** *N*-hydroxyethyl-1-deoxynojirimycin (Miglitol), **4** DL-*threo*-1-Phenyl-2-decanoylamino-3-morpholino-1-propanol (PDMP), **5** DL-*threo*-1-Phenyl-2-palmitoylamino-3-morpholino-1-propanol (PPMP).

**Figure 2:**
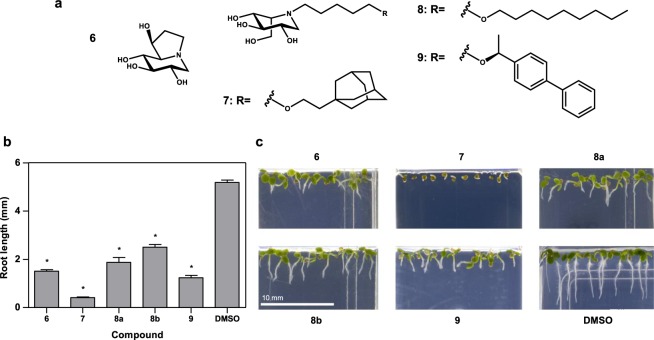
Root growth inhibitors identified from an iminosugar compound library screened against Arabidopsis. (**a**) The structures of compounds identified as causing significant inhibition of primary root growth in germinating Arabidopsis seedlings. **6** Castanospermine, **7** l-*ido*-AEP-DNJ, **8** l-*ido*-5-(nonyloxy)pentyl-DNJ, **9** l-*ido*-5-[(1S)-1-(4-phenylphenyl)ethoxy]pentyl-DNJ. (**b**) Arabidopsis primary root length measurements in the presence of 10 µM inhibitor after 7 days. Values of root length shown are the mean value for 6–10 seedlings. Error bars represent SEM. Values marked with asterisks are statistically significant from the control (DMSO) values (t-test; *P < 0.05). (**c**) Representative images of the phenotype observed for the given inhibitors.

**Figure 3:**
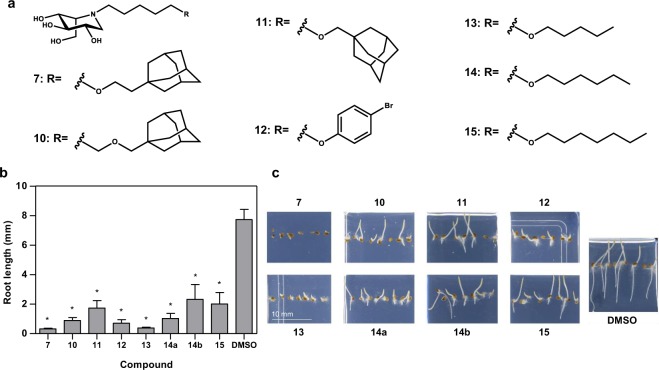
Root growth inhibitors identified from an iminosugar compound library screened against Tef. (**a**) The structures of compounds capable of inhibition of Tef primary root growth following seed germination. **7** l-*ido*-AEP-DNJ, **10** l-*ido*-AMH-DNJ, **11** l-*ido*-AMP-DNJ, **12** l-*ido*-5-(bromophenyloxy)pentyl-DNJ, **13** l-*ido*-5-(pentyloxy)pentyl-DNJ, **14** l-*ido*-5-(hexyloxy)pentyl-DNJ, **15** l-*ido*-5-(heptyloxy)pentyl-DNJ. (**b**) The primary root length measurements of Tef grown in the presence of 10 µM inhibitor after 3 days. Values of root length shown are the mean value for 4–8 seedlings. Error bars represent SEM. Values marked with asterisks are statistically significant from the control (DMSO) values (t-test; *P < 0.05). (**c**) Representative images of the phenotype observed for the given inhibitors.

Iminosugars are a group of naturally occurring sugar-like molecules in which the endocyclic ring oxygen is replaced by a nitrogen atom^6^. Several of these compounds are utilised as drugs to treat diabetes and errors in glycosphingolipid metabolism^7,8^. Furthermore, iminosugars have been thoroughly studied in mammalian systems due to their potential as therapeutic agents to treat conditions including cancer, viral infection and genetic disorders of carbohydrate metabolism^6^. We have previously investigated the scope for iminosugars as tools to dissect metabolic pathways involved in the germination and growth of crop plant species, with focus on starch metabolism^9^. *N*-Hydroxyethyl-1-deoxynojirimycin (Miglitol, Fig. 1-**3**) has been tested on germinating wheat seeds, resulting in lower levels of α-glucosidase activity, accumulation of maltose and a decrease in the rate of starch degradation^10^. Taking this study further, a selection of iminosugars and iminosugar-like molecules were screened against recombinant barley α-glucosidase (Agl97) to identify suitable inhibitors^11,12^. Three compounds (1-deoxynojirimycin [DNJ], *N*-butyl-1-deoxynojirimycin [*N*-Bu-DNJ] and Miglitol, Fig. 1-**1**, **2** & **3**) gave over 80% inhibition of α-glucosidase activity at 10 µM. These inhibitors were further tested on germinating barley and shown to inhibit α-glucosidase activity, decrease grain starch metabolism and retard root growth. RNAi knockdown of the sole endosperm α-glucosidase indicated that this enzyme has little effect on starch degradation or growth. In detached embryos treated with exogenous sugars and inhibitors the compounds still caused stunted root growth, thus revealing the existence of unidentified iminosugar targets^11^.

It has been proposed that the inhibition of seedling growth caused by DNJ in the presence of exogenous glucose may be due to inhibition of glycoprotein processing^11^. DNJ and related glucosidase inhibitors have been shown to inhibit the *N*-glycan processing enzymes glucosidase I and II, in mammalian systems^13–15^. Plant knockout mutants in these enzymes are lethal indicating important roles in growth and development^3,16–20^. Iminosugar derivatives including castanospermine and *N*-methyl-DNJ have been shown to impact on glycoprotein processing within the leaves of radish seedlings; inhibition of *N*-glycan trimming was proportional to inhibition of growth^21,22^. It is not yet clear that the DNJ-induced root growth inhibition is directly linked to the inhibition of *N*-glycan trimming glucosidases in plants. There is potential that synthesis of the dolichol-linked *N*-glycan precursor may be affected by DNJ^23^. Cell wall-associated glycan modifying enzymes may also be inhibited^24^. Recently the link between starch and cell wall metabolism in germinating barley has been dissected using the iminosugar 1,4-dideoxy-1,4-imino-l-arabinitol (LAB), an inhibitor of arabinoxylan-hydrolase enzymes^25^. Other potential targets for iminosugars in plants include the carbohydrate binding proteins involved in cellular regulation and glycolipid processing enzymes^4,26^.

**Figure 4:**
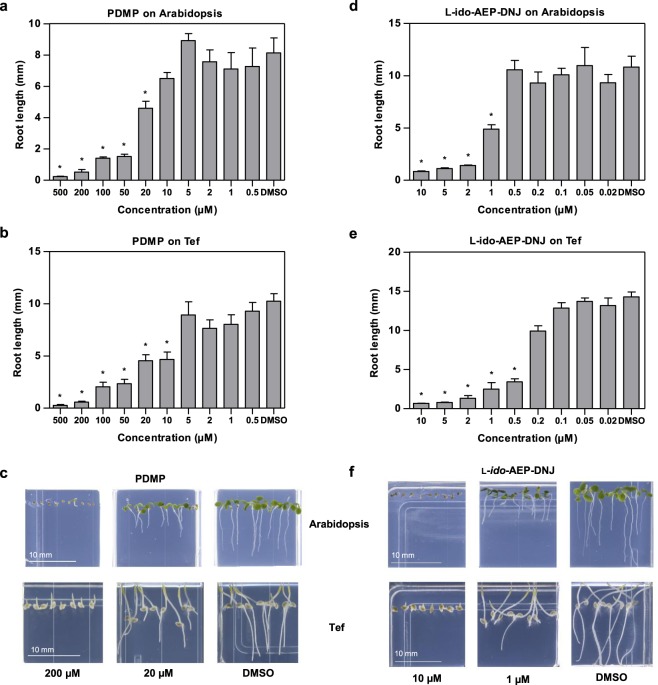
The iminosugar l-*ido*-AEP-DNJ generates a root growth inhibition phenotype similar to the known glucosylceramide synthase inhibitor PDMP. Primary root length measurements in the presence of varying PDMP concentrations. (**a**) Arabidopsis seedlings grown for 7 days. (**b**) Tef seedlings grown for 3 days. (**c**) Representative images of the phenotype observed when Arabidopsis and Tef are grown in the presence of PDMP. Primary root length measurements in the presence of varying l-*ido*-AEP-DNJ concentrations. (**d**) Arabidopsis seedlings grown for 7 days. (**e**) Tef seedlings grown for 3 days. (**f**) Representative images of the phenotype observed when Arabidopsis and Tef are grown in the presence of l-*ido*-AEP-DNJ. Values of root length shown are the mean value for 4–12 seedlings. Error bars represent SEM. Values marked with asterisks are statistically significant from the control (DMSO) values (t-test; *P < 0.05).

Iminosugars are used in humans to treat lysosomal storage disorders which involve defective glycosphingolipid glycosyl hydrolases^27^. Gaucher’s disease is caused by mutations in the gene encoding β-glucocerebrosidase (glucosylceramidase, GCase) leading to an inactive or partially active enzyme which results in glucosylceramide (GlcCer) accumulation (Fig. 1)^28^. This build-up of GlcCer leads to symptoms such as neurological dysfunction, osteoporosis and hepatomegaly^29^. Glucosylceramide synthase (UDP-glucose: ceramide β-1,4-glucosyltransferase, GCS) is responsible for GlcCer synthesis, catalysing the transfer of a glucose moiety from UDP-glucose to ceramide (Cer) (Fig. 1)^30^. GCS is an established target for the treatment of Gaucher’s disease; a process termed substrate reduction therapy involves inhibition of GCS, leading to decreased accumulation of GlcCer *in vivo*^31^. There are two archetypal GCS inhibitors: PDMP^32^ and *N*-butyl-deoxynojirimycin (*N*-Bu-DNJ) (Fig. 1-**2**, **4**)^33^; most reported inhibitors are related to these scaffolds. Work to increase iminosugar potency and selectivity has focused on human GCS and other human glycoprocessing enzymes. A large collection of *N*-alkyl substituents, as well as structural and stereochemical analogues of the d-*gluco*-configured *N*-Bu-DNJ have been synthesised to analyse how inhibitor structure alters selectivity for GCS over GCase and other glucosidases^1,34–38^.

The roles played by Cer and GlcCer are well studied in mammalian and fungal systems, yet fall behind in plant systems^39^. However, GlcCer has been implicated in plant cellular organization, lipid microdomain architecture, membrane fluidity, vesicle formation, pollen development, cell wall biosynthesis and degradation, and response to biotic and abiotic stresses^40–42^. Mutants of GCS have been reported in Arabidopsis^5^. Null mutants were viable as seedlings, with strongly reduced size and altered Golgi morphology, but failed to develop beyond seedling stage. RNAi suppression of the single gene encoding GCS enabled the generation of viable plants with ≤2% of wild type GlcCer levels indicating that only low levels of this enzyme are needed for cell differentiation, organ development and seedling establishment^5^. It remains unclear why GCS activity is essential for development and why its absence cannot be complemented by other metabolic pathways.

Chemical genetics, the use of small molecule inhibitors in place of loss-of-function mutations, offers the opportunity to avoid the lethal effect of genetic knockout of essential genes^43,44^. Compounds can also be applied across species, therefore avoiding the complexities associated with traditional genetic knockout techniques in crop species, which are exacerbated by large genomes, polyploidy, gene redundancy, and slow generation times^45,46^. Cyclophellitol aziridine based probes have proven useful to reveal the importance of glycosidases in several model and crop plant systems^47–49^. Inhibitors of recombinant barley β-amylase have also been identified and used to probe the catalytic mechanism of this enzyme^50^. To identify novel targets for iminosugars in plants, we investigated the effect of iminosugar derivatives on seedling root growth. Herein we report a screen of a library of *N*-substituted DNJ analogues against germinating seeds of Arabidopsis and the cereal Eragrostis tef (Tef). Several potent inhibitors of primary root growth were identified. Further analysis of the strongest inhibitor demonstrated that this root phenotype was linked to the inhibition of GCS and depletion of glucosylceramide relative to ceramide levels.

## Results

### Identification of novel DNJ-derivatives capable of inhibition of Arabidopsis root growth

To identify novel inhibitors and determine the effect that derivatives of DNJ have on germination and seedling growth, we blind-screened a collection of 390 iminosugar derivatives, at a concentration of 10 μM, in agar plate based Arabidopsis root growth assays. The compounds in this library were designed to be active against glycosidases of medical relevance and have shown promise as inhibitors of several important mammalian targets. A variety of iminosugar stereochemistry and *N*-substitution were represented to enable the analysis of structure-activity relationships (see Materials and Methods and Supplementary section for details). From this screen, five compounds were identified as having a strong inhibitory impact on root growth (Figs 2 and S1). A threshold of 50% inhibition of growth, compared to control, was used to define hit compounds, which produced a hit rate of 1.3%.

Four l-*ido*-configured sugars were identified from the screen, along with castanospermine, **6**, a known potent inhibitor of root growth in dicots^51^. Compound **7** (l-*ido*-AEP-DNJ) gave almost complete inhibition of primary root growth (92% inhibition of root length); germination was not inhibited, as radicle emergence could be seen (Fig. 2c). Three other l-*ido*-configured compounds were identified in the screen (Fig. 2a, **8a**, **8b** & **9**). Compound **8** was present in duplicate (**8a** and **8b**) within the library and gave 64% and 52% root growth inhibition. The 12% variation between these two compounds highlights the variability between different plates within the assay, but also acts as an internal control for the screen, indicating that duplicates gave comparable results. Compound 9 gave 76% root growth inhibition. Notably, all the inhibitory l-*ido* compounds had long, lipophilic carbon chains as indicated by positive partition coefficient (LogP) values (Table S1). The different arrangement of carbon chains (alkyl, aromatic and 3D) represented in the hit compounds (Fig. 2a) indicate that the arrangement is less important than chain length and lipophilicity.

Extending the threshold to 40% inhibition of growth, nine d-gluco-configured DNJ variants, including one duplicate, were also identified as root growth inhibitors (Figs S1 and S2). These compounds contain large lipophilic aromatic *N*-substituent groups. No other l-*ido*-configured compounds (of a total of 92) showed significant root growth inhibition at 10 µM, including pentyl- l-*ido*-DNJ and l-*ido*-AMP-DNJ (**10**), which differs from l-*ido*-AEP-DNJ (**7**) by having one fewer carbons (Fig. S3a) and is one of the lead compounds for inhibition of mammalian GCS^36^. Other compounds that showed no inhibition at 10 µM include *N*-Bu-DNJ, *N*-pentyl-DNJ and d-*gluco*-configured AMP-DNJ (Fig. S3a). This is perhaps surprising given the potential impact these compounds may have on glycoprotein processing but indicates the potency of the identified inhibitors compared to other iminosugars.

### l-*ido*-Configured iminosugars are root growth inhibitors of the cereal *Eragrostis tef*

To determine whether the identified hit compounds have similar effects on root growth of monocots as in dicots, we sought a monocot alternative for screening. Larger cereals (wheat, barley, rice and maize) are not amenable to medium-throughput assays as the combination of grain size and the quantity of inhibitor required to elicit a response is too high for practical use. We identified Tef to be a suitable size for use in the same screening process as Arabidopsis. Tef is a cereal rich in minerals, protein and starch, indigenous to the horn of Africa. The main area of cultivation is Ethiopia, where it is utilised for bread production and also as the base for a traditional Ethiopian beer called Tella. Recently, Tef has been employed in a robotic phenotyping platform with application in chemical genetic screening^52^. Tef grains utilise starch as their primary energy reserve and they have the necessary starch degrading enzymes, making it a convenient, fast germinating and growing model for other cereals which utilise starch as their main grain energy reserve^53^. Furthermore, the Tef genome sequence has been sequenced, enabling genomic analysis and potential genetic validation of chemically-identified carbohydrate metabolism targets^54^.

We screened the library of 390 iminosugar derivatives against Tef at a concentration of 10 μM. Eight compounds were identified as hits, using a threshold of 70% root growth inhibition compared to control (Fig. 3). This generated a 2% hit rate. In keeping with the Arabidopsis screen, all hit compounds identified were l-*ido*-configured iminosugars (Fig. 3a), although the *N*-substituents were more varied. Compounds **7**, **10** and **11** (96%, 88% and 78% inhibition, respectively) all possess an adamantyl group with different length linkers. Compound **12** contains a lipophilic bromophenyl group and inhibited growth by 91%. Four of the compounds identified had alkoxy groups with lengths differing by 2 carbons: **13**, **14** (**a** and **b**) and **15**, these compounds inhibited growth by 95%, 87%, 70% and 74% respectively. Two of these compounds **14** (**a** and **b**) have the same structure thus acting as an internal control. Castanospermine was not identified as an inhibitor of Tef root growth; this confirms the different response to this compound between monocots and dicots that is known to exist^51^. The molecular basis of this difference remains to be established.

Other l-*ido*-configured iminosugars, including pentyl- l-*ido*-DNJ, showed no significant root growth inhibition against Tef (Fig. S3b). d-*Gluco*-configured *N*-pentyl-DNJ and *N*-Bu-DNJ gave no root growth inhibition, however, AMP-DNJ caused partial inhibition at a concentration of 10 µM. The level of inhibition was above the 70% inhibition threshold used to select strong inhibitory compounds. Several other d-gluco-configured compounds showed between 50–70% inhibition. This inhibition indicates the susceptibility of cereals to d-gluco-configured iminosugars, as also observed with DNJ^11^.

Comparison of the results from the Arabidopsis and Tef screens highlights several similar features: all the hits are l-*ido*-configured iminosugars and possess long lipophilic *N*-substituents, containing adamantyl (**7**, **10** & **11**) or alkyl (**8**, **13**, **14** & **15**) functional groups. Both the Arabidopsis and Tef screens identified compound **7** (l-*ido*-AEP-DNJ) as the strongest inhibitor of primary root growth, making this a leading candidate for further analysis.

### The identified l-*ido*-configured inhibitors are more potent than established non-iminosugar glucosylceramide synthase inhibitors which impact on Arabidopsis, Tef and barley root growth

Given that several of the compounds identified from our screens are known inhibitors of human GCS and the most potent inhibitor identified in both Arabidopsis and Tef screens (l-*ido*-AEP-DNJ) differs in only one carbon to a lead compound for mammalian GCS inhibition (l-*ido*-AMP-DNJ)^1,36^, we hypothesised that the root growth inhibition phenotype may be due to inhibition of plant GCS. To test this hypothesis, commercially available non-iminosugar inhibitors of mammalian GCS (PDMP and PPMP, **4** & **5**) were tested at a range of concentrations against Arabidopsis and Tef to compare the phenotype generated.

**Figure 5:**
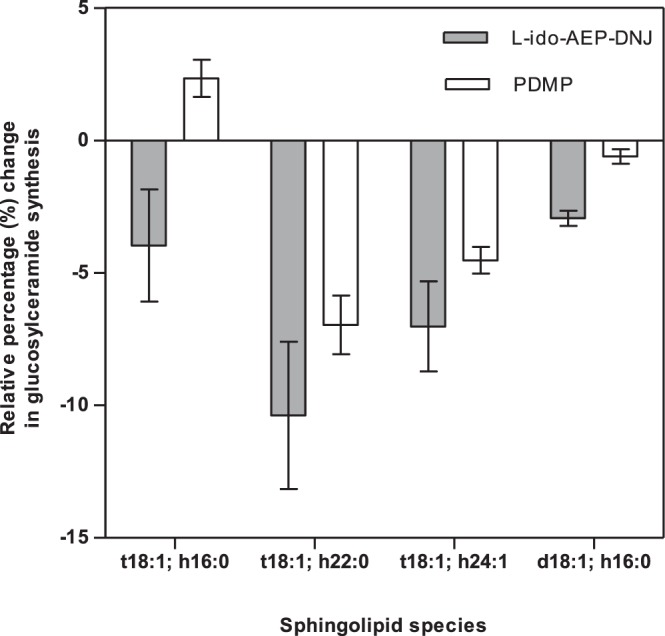
Changes in glucosylceramide levels relative to respective ceramide in inhibitor treated seedlings analysed using LC-MS/MS. LC-MS/MS analysis of Cer and GlcCer in extracts from Arabidopsis grown in the presence of l-*ido*-AEP-DNJ (1 µM) and PDMP (10 µM); levels of Cer and GlcCer in inhibitor treated plants were compared to control samples and the difference expressed as percentage change in GlcCer synthesis. Seedlings grown for 26 days. Data represent average of three biological replicates, error bars indicate SEM. Regarding ceramide nomenclature, details on the long chain base are given followed by fatty acid chain, separated by a semicolon. Chains are designated h, d or t corresponding to monohydroxy, dihydroxy or trihydroxy species. Carbon chain length is given, followed by a number corresponding to the level of unsaturation within the carbon chain. i.e. t18:1; h16:0 represents a trihydroxy long chain base with an 18 carbon chain with one unsaturated bond attached to a monohydroxylated fatty acid chain of 16 carbons with no unsaturation.

At concentrations above 200 μM, PDMP elicited a similar root growth phenotype to those seen with Arabidopsis and Tef treated with the hit l-*ido*-DNJ derivatives at 10 μM. We found PDMP to give much stronger inhibition compared to the more lipophilic PPMP (Figs 4a–c and S4a,b); PPMP gave no significant inhibition of root growth with Arabidopsis or Tef whereas PDMP showed significant inhibition of root growth at concentrations of 20 µM for Arabidopsis and 10 µM for Tef. The difference in alkyl chain length has an impact on the level of root growth inhibition: PPMP has a 15 C alkyl chain whereas PDMP has a 9 C alkyl chain (Fig. 1, **4** and **5**). There is a possibly that PPMP is too lipophilic. The prediction of logP values for the inhibitors used in this study (Table S1) indicate that PPMP is very lipophilic and may have problems with bioavailability meaning the compound may not be able to enter cells. When PDMP was tested on germinating barley (Fig. S5) a concentration of 500 μM was required to elicit a significant phenotype after 10 days. This is fifty-fold higher than the concentration required for significant inhibition in the small cereal Tef and indicates the high concentration of compound that would be required to elicit responses in larger species.

We further analysed the dose-response relationship of l-*ido*-AEP-DNJ on Arabidopsis and Tef (Fig. 4d–f) and compared this to the results using PDMP. IC_50_ values were calculated for root growth inhibition (Table 1, Fig. S6); l-*ido*-AEP-DNJ was 20-fold more potent than PDMP. A 10 µM concentration of l-*ido*-AEP-DNJ gave almost complete inhibition of root growth, however germination rate was not dramatically affected (Fig. S7).

**Table 1:** IC_50_ values for PDMP and l-*ido*-AEP-DNJ inhibition of Arabidopsis and Tef root growth.

IC_50_ values (µM)	PDMP	L-*ido*-AEP-DNJ
Arabidopsis	22.02	0.99
Tef	17.66	0.29

Calculated from graphs shown in Fig. S7, plotted using root growth data taken from Fig. 4.

### Plant glucosylceramide synthase is different in sequence to the mammalian protein and is present in all plant species analysed

To identify Arabidopsis proteins with similarity to human GCS (HsGCS, UniProt: Q16739), which may represent potential targets for l-*ido*-DNJ derivatives, we utilised a domain enhanced lookup time accelerated BLAST (DELTA-BLAST) search against the Arabidopsis thaliana genome. This search indicated that HsGCS shares maximum homology with Arabidopsis GCS (TAIR: At2g19880), the only sequence with over 30% identity (31%) and a coverage of 33%. Domain and secondary structure analysis indicates that both plant and human GCS proteins contain an *N*-terminal transmembrane helix, a cytosolic domain including the putative active site and C-terminal transmembrane/membrane associated helices. The 120 amino acid region of homology lies within cytosolic catalytic domain. The only other sequence identified with an expect (E) value below 0.001 is a cellulose synthase-like C12 protein (24% identity, 54% coverage, TAIR: At4g07960).

We aligned the Arabidopsis and Tef GCS protein sequences with those from other eukaryotes (*H. sapiens, D. melanogaster, C. elegans, P. pastoris* and *C. albicans*) (Fig. S9) to compare sequence similarities and determine whether any conserved motifs important for inhibition by l-*ido*-DNJ derivatives could be identified. The overall identity between the plant and non-plant sequences was low, giving between 8–14%. Arabidopsis and Tef GCS show a high sequence similarity (73%), indicating conservation between these species. Sixteen residues are 100% conserved throughout all eukaryotic GCSs (indicated with (∙) in Fig. S8). These residues include the DDD motif which has been shown to be essential for substrate binding and catalysis in human GCS^30,55^; however, the plant sequences lack the RXXRW motif that is associated with the DDD motif and is essential for function in human GCS^55^. The significance of the absence of this motif in the plant sequence remains to be determined.

To determine the prevalence of GCS in plant species, we identified GCS sequences by BLAST analysis and performed a ClustalW multiple sequence alignment for fifteen selected common crop plant species (Fig. S9). This analysis indicated that the gene encoding GCS is present in all species studied. Alignment shows that there is high identity (69–99%) between the selected higher plant sequences. Three homoeologues were identified in the wheat genome, corresponding to the A, B and D genome copies.

### LC-ESI-MS/MS analysis of inhibitor treated Arabidopsis indicates a decrease in glucosylceramide relative to ceramide

We reasoned that if the inhibitory effects on root growth were due to inhibition of GCS then seedlings would exhibit an accumulation of Cer with an associated decrease in GlcCer. We therefore utilised *in vivo* analysis of Cer and GlcCer and measured their relative levels in Arabidopsis seedlings treated with PDMP and l-*ido*-AEP-DNJ. Based on published sphingolipidome profiling from Arabidopsis^56^, we selected four major Cer and GlcCer pairs (Fig. 5) (t18:1; h16:0, t18:1; h22:0, t18:1 h24:1, t18:1 h16:0) for monitoring in plant extracts using LC-ESI-MS/MS. Arabidopsis seedlings were grown for 26 days in the presence of the inhibitors and sphingolipids were extracted from the plant tissue. Seedlings growing in the absence of inhibitors were used as a control. The levels of diagnostic Cers and the corresponding GlcCers were compared between control and inhibitor treated plants; the difference was expressed as a relative percentage change in GlcCer levels (Fig. 5).

Both l-*ido*-AEP-DNJ (1 µM) and PDMP (10 µM) caused a relative decrease in GlcCer levels. l-*ido*-AEP-DNJ and PDMP treated samples both generated similar patterns of reduction in GlcCer levels with t18:1; h22:0 and t18:1; h24:1 being the two most strongly impacted Cer:GlcCer pairs. When treated with l-*ido*-AEP-DNJ the levels of GlcCer relative to Cer in the diagnostic pairs is decreased by 2–10%, depending on the specific sphingolipid. l-*ido*-AEP-DNJ gave stronger reduction in GlcCer synthesis than did PDMP, indicating the increased potency and likely increased selectivity of l-*ido*-AEP-DNJ for GCS, consistent with the root phenotypic screen data. PDMP treated Arabidopsis extracts show a reduction in GlcCer for three out of the four lipid species tested, although t18:1; h16:0 gave a slight increase in GlcCer levels: the reason for this is unclear.

## Discussion

This study aimed to identify novel iminosugar inhibitors of seedling root growth by screening a library of *N*-substituted DNJ analogues against germinating seeds of Arabidopsis and the cereal Eragrostis tef. Four out of five hits from our Arabidopsis screen and all eight hits identified with Tef are l-*ido*-configured iminosugars. The single other hit compound was castanospermine, a known inhibitor of *N*-glycan processing glucosidases. The effects of castanospermine on Arabidopsis and Tef reported herein highlight the difference in response that can occur between monocots and dicots. This phenomenon has previously been noted^51^ yet the underlying biochemical explanation has not been established. Furthermore we observed a difference in the potency of the iminosugars on Arabidopsis and Tef, as indicated by the threshold used to elicit a hit rate below 2%, i.e. 50% for Arabidopsis but 70% for Tef. The reason for the difference in susceptibility is unclear but may be linked to the differences in carbohydrate metabolism between Arabidopsis and Tef; there is potential for the iminosugars to interfere with starch metabolism in germinating Tef. These differences highlight that Tef is a useful alternative to Arabidopsis for screening for cereal-specific inhibitor effects.

The identification of only l-*ido*-DNJ derivatives from a library containing different iminosugar stereochemistries indicates that this configuration is important for potent inhibition of root growth. Another recurring theme within the identified compounds is the presence of large, lipid soluble, *N*-substituents. The *N*-substituents are likely important for compound uptake, enabling diffusion across cell membranes and/or correct presentation within a membrane for interaction with a membrane bound protein. Lipophilicity has been shown to be important for compound uptake in plants, however high lipophilicity (high logP) can hinder uptake through roots and seed coats^57^. The fact that only specific *N*-substituted l-*ido*-configured iminosugars were identified as strong root growth inhibitors indicates that the inhibition is via a specific mechanism and not simply the result of a detergent-like effect, which might interfere with membrane structure and perturb growth.

The lead compound identified from the chemical genetic screens, l-*ido*-AEP-DNJ, is one carbon larger than the potent mammalian GCS inhibitor l-*ido*-AMP-DNJ (*N*-5-[adamantane-1-yl-methoxy]pentyl- l-*ido*-DNJ)^1,36^. This led us to hypothesise that root growth inhibition is linked to plant GlcCer metabolism. Phenocopying the effect of l-*ido*-AEP-DNJ with the known GCS inhibitor PDMP revealed a similar concentration-dependent stunted root growth phenotype. Several studies have investigated the effect of PDMP on plants using N. benthamiana leaves or cultured Arabidopsis root cells^58,59^. When treated with PDMP, Golgi morphology and protein secretion are perturbed^58^, PDMP also affects vacuole morphology^59^. To date, there is no literature precedent for the effect of iminosugars on GCS in planta. Our results indicate that in the presence of l-*ido*-AEP-DNJ or PDMP the levels of four diagnostic GlcCers decreased relative to Cer levels, when compared to untreated plants, with one exception of PDMP causing a slight increase in GlcCer synthesis (t18;1: h16;0) relative to the corresponding Cer. This is consistent with partial inhibition of GCS. The moderate extent of decrease in conversion of Cer in the corresponding GlcCer (Fig. 5) arises from the fact that the concentration of inhibitors used to elicit a root growth inhibition phenotype was relatively low. Higher inhibitor concentrations would reduce plant growth to such extent that insufficient tissue would be generated for extraction. However, even in the presence of low concentrations of inhibitor, a reduction in GlcCer activity could be detected.

Mutants of the single gene encoding GCS have been generated and analysed in Arabidopsis^5^. Null mutants failed to develop beyond seedling stage. The inhibited root growth phenotype observed in these mutants supports our proposal that the root growth inhibition caused by l-*ido*-AMP-DNJ is mediated by GCS inhibition. Glycosyl inositol phosphoryl ceramide (GIPC) content was increased in GCS mutant lines. It is not clear whether our chemical perturbation of GlcCer synthesis impacts on other sphingolipid species. It is possible that changes in GlcCer levels are compensated for by changes in GIPC in a similar manner to the genetic mutant. RNAi plants with ≤2% of wild type GlcCer levels were viable, indicating that only small amounts of GCS are essential for developmental processes. GCS RNAi lines with up to 85% reduction in GCS transcripts show increased susceptibility to PDMP in root growth assays^60^. It is likely that GlcCer mediated interference with the membrane structure of subcellular compartments is responsible for the inhibition of root growth. Indeed, Melser *et al*. have shown that alteration in the transport and degradation of PIN proteins, which are responsible for the transport of auxin across membranes, may be responsible in some part for root growth inhibition in PDMP treated seedlings^60^. The nature of how cellular processes such as cell growth, cell division, hormone signalling and signal transduction pathways are affected by l-*ido*-AEP-DNJ and PDMP remains to be dissected and presents avenues for future experimentation.

The specificity of l-*ido*-AEP-DNJ is yet to be fully addressed in plants, we cannot rule out that other targets exist for this compound. It is possible that this compound shows partial inhibitory activity towards enzymes related to cellulose synthesis, as these glycosyltransferase proteins have some similarity to GCS. Furthermore, there is a possibility that the l-*ido*-compounds show activity against both GCS and GCase. GCase has not been characterised in plants, a putative activity has however been identified in bean and squash membrane extracts^61^. Studies using the mammalian enzymes show l-*ido*-AMP-DNJ is twenty times more selective towards GCS over GCase when compared to d-gluco-AMP-DNJ, it is likely that l-*ido*-AEP-DNJ will follow this trend^38^. PDMP has been shown to have alternative, non-GCS related, effects in mammalian studies, including inhibition of other enzymes involved in glycosphingolipid metabolism^62,63^ impacting on organellar behaviour^64^ and interfering with calcium homeostasis^65^. The impact of PDMP on calcium homeostasis has been ruled out in plants^58^, though it remains to be shown whether the effect of PDMP is due to increased Cer levels alone or combined with an alternative mechanism.

The bioinformatic analysis carried out herein indicates that the Arabidopsis gene with the highest homology to the Human GCS is Arabidopsis GCS. This gene is conserved across all plant species analysed, including Arabidopsis and Tef. This information, alongside the LC-ESI-MS/MS data presented herein and the similarity of the phenotype compared to PDMP, a known GCS inhibitor, has led us to propose that GCS is the likely target responsible for the root growth inhibition seen in l-*ido*-AEP-DNJ treated plants. Our results indicate that l-*ido*-AEP-DNJ is a potent inhibitor of seedling root growth and represents a valuable tool that can be used to study the function of GCS at developmental stages. This chemical inhibitor can be added at specific time points, in a titratable manner, to elicit a response. It offers the potential to study the role of Cer and GlcCer in crop species that would otherwise require lengthy genetic manipulation strategies. The identification of three homoeologues in the wheat genome indicates the complexity that would be involved in genetic manipulation of GCS in polyploid crop species. The inhibitors identified herein offer a potential alternative to genetic manipulation, and will enable a better understanding of how GCS and GlcCer metabolism modulates plant root growth and responses to environmental stimuli.

## Materials and Methods

### Iminosugar library and control inhibitors

The iminosugar library consisted of 390 compounds which were synthesised for several studies of mammalian glycosidases and glycosyltransferases^1,34–38,66^. A range of hexose stereochemistries are represented within the library including d-*gluco*, l-*ido*, d-*galacto* and l-*altro* configurations. Most compounds are 1-deoxy iminosugars although a number are 1,2 dideoxy and 1,2,3 trideoxy, tetrahydropyridineol derivatives. The majority of structures within the library are d-gluco (121) or l-*ido* (92) iminosugars with *N*-alkyl substitutions or modifications at the anomeric carbon. The substituents include alkyl, alkoxyadamantyl, alkoxybiphenyl, and isoprene moieties containing different carbon chain lengths. Some compounds were further substituted at the 1 position with alkyl and adamantly groups. 1,4-Dideoxy-1,4-imino-L-arabinitol (LAB) and castanospermine derivatives were also present. The full structures of all compounds represented in this library are given in the supplementary file. The neat compounds were obtained in a 96-well format and DMSO was added to produce working concentrations of 10 mM. *N*-[(R*,R*)-2-hydroxy-1-(4-morpholinylmethyl)-2-phenylethyl]-hexadecanamide hydrochloride (PPMP) and *N*-[(R*,R*)-2-hydroxy-1-(4-morpholinylmethyl)-2-phenylethyl]-decanamide hydrochloride (PDMP) were purchased from Sigma-Aldrich (now Merck) and Calbiochem (now Merck), respectively.

### Plant root growth assays

25-Well sterile plastic plates (Sterilin, UK) containing compounds were prepared by combining 2 mL half-strength Murashige and Skoog (MS) medium supplemented with 1% agar (Arabidopsis) or 2 mL 1% agar (Tef) with a 2 µL inhibitor solution in DMSO. Control seedlings were grown in the presence of 0.1% DMSO.

All seeds were surface sterilised and rinsed. Arabidopsis and Tef were plated out on to agar. 10–12 Arabidopsis seeds (accession Col-0) or 8 Tef grains (var. Trotter or Tsedey [DZ-Cr-37]) were placed in a straight line across each well. Plates were sealed with micropore tape (3M), stratified for 2 d at 4 °C and then incubated vertically. Ten barley grains (var. Tipple) were plated out on to two layers of filter paper pre-soaked with 2 mL H_2_O containing 2 µL of inhibitor solution in DMSO, in a petri dish. Ten Brassica rapa seeds were plated out on petri dishes containing 5 ml half-strength MS medium supplemented with 1% agar with the addition of 5 µL inhibitor solution in DMSO.

Arabidopsis and Brassica rapa were incubated at 22 °C in a controlled environment room (16 h light: 8 h dark, 22 °C, 250 µmol photosynthetically active radiation m^−2^ s^−1^) for 10 d. Tef and barley were incubated in a dark Hybaid incubator (Thermo) at 17 °C within a cold room for 3 d and 10 d respectively.

For 25-well plates, roots were imaged by photography of the plate, then root length was manually measured using ImageJ software. Roots of petri dish grown seeds were manually measured using a ruler.

### Sample preparation for LC-MS/MS analysis

Arabidopsis seedlings were grown as given above in the presence of l-*ido*-AEP-DNJ (1 µM) or PDMP (10 µM). Control seedlings were grown in the presence of 0.1% DMSO. Seedlings were grown for 26 d to produce sufficient plant tissue. Approximately 30 mg of freeze-dried Arabidopsis tissue was weighed accurately and the plant material was homogenised in a ceramic mortar together with 3 mL of extraction solvent (lower phase of isopropanol/hexane/water 55:20:25 v/v/v)^56^. The sample was transferred into a 10 mL glass tube. The mortar and pestle were rinsed with an additional 2 mL of the extraction solvent which was added to the homogenate. The tube was capped and incubated at 60 °C for 15 min. The sample was centrifuged (500 × g, 4 °C, 10 min) and the supernatant was collected. The pellet was re-suspended in 3 mL of the extraction solvent and extracted again by incubation at 60 °C for 15 min and centrifugation as before. The supernatants were combined in a glass vial and evaporated to dryness in a centrifugal evaporator (Genevac EZ-2). The residue was de-esterified by adding 2 mL of 33% methylamine solution in ethanol/water (7:3 v/v) and incubating at 50 °C for 1 h. The sample was evaporated to dryness in a centrifugal evaporator (Genevac EZ-2). The residue was dissolved with vortexing in 1 mL of methanol with 2 mM ammonium formate and 0.1% v/v formic acid. The sample was centrifuged (16873 × g, room temperature, 10 min) and the supernatant was filtered with a disc filter (0.45 µm, PTFE) and stored at −20 °C until analysis. Analysis of 3 biological replicates was performed.

### Sphingolipid standards

Synthetic Cer and GlcCer standards: Cer (d18:1; c12:0), GlcCer (d18:1; c12:0) and GlcCer extracted from soy were purchased from Avanti Polar Lipids, Inc. (Alabaster, AL). The soy GlcCer extract contains (>98%) mainly GlcCer (d18:2; h16:0). Acid hydrolysis (HCl) of the soy GlcCer afforded a mixture containing Cer (d18:2; h16:0) as the principal component.

### LC-MS/MS

LC-MS/MS analysis of sphingolipids was performed on a Xevo TQ-S tandem quadrupole mass spectrometer (Waters) operated in multiple reaction monitoring (MRM) mode. The mass spectrometer was coupled to an Acquity UPLC. Online chromatographic separation was achieved using reversed phase C18 column Kinetex XB-C18 (100 × 2.1 mm, 2.6 µm) equipped with KrudKatcher Ultra in-line filter. A multistep gradient elution at 40 °C and flow rate 300 µl/min with mobile phase B (methanol with 2 mM ammonium formate and 0.1% formic acid) against mobile phase A (water with 2 mM ammonium formate and 0.1% formic acid) was employed^67^. The elution started with 50% mobile phase B for 1.2 min, followed by gradient to 80% B over 1.2 min, then a gradient to 100% B over 8 min, held isocratic for 5 min, then returned to 50% B in 0.1 min and held for a further 3.0 min to equilibrate the LC column. The total analysis time was 18.5 min but the column flow in time-window from 0 to 3 min was discarded. ESI-MS/MS analysis was performed in positive ion mode using a source with a capillary voltage of 1.5 kV, 500 °C desolvation temperature, 1000 l.hr^−1^ desolvation gas, 150 l.hr^−1^ cone gas, and 7 bar nebulizer pressure. MRM transitions, optimised cone voltage and collision energy values for GlcCer and Cer standards in positive ESI mode were generated using IntelliStart software (Table S2). Stock solutions of sphingolipid standards (1 mg/mL) were prepared in chloroform-methanol (1:4, v/v)^67^ filtered through 0.45 µm PTFE disk filter and stored at −20 °C. The stock solutions were diluted to concentration 10 µM in methanol containing 2 mM ammonium formate and 0.1% formic acid. Samples (10 µM) were introduced at 10 µl/min combined with a flow from the HPLC pump typical of an LC run. Once LC retention times of standards were established (Table S2), the mass transitions were collected in time-windows centred on the relevant peaks, to avoid collecting excessive numbers of transitions simultaneously. MRM transitions for diagnostic pairs of Arabidopsis thaliana Cers and GlcCers were adapted from Markham and Jaworski^56^. These include Cers (t18:1; h16:0), (t18:1; h22:0), (t18:1; h24:1) and (d18:1; h16:0) and their corresponding GlcCers (Table S2). Limit of detection was determined to be 2 pmol on column using a serial dilution from 1 nM to 10 µM of Cer (d18:1; c12:0). MassLynx software (Waters) was used to collect, analyse and process data.

## Electronic supplementary material

Supplementary Information and Data

Supplementary Information Library Structures
