# Supplementary material for: A chemical genetic screen reveals that iminosugar inhibitors of plant glucosylceramide synthase inhibit root growth in Arabidopsis and cereals
Source: Sci Rep. 2018 Nov 6;8:16421. doi: 10.1038/s41598-018-34749-1 (PMC6219604; doi:10.1038/s41598-018-34749-1)
Supplement: Supplementary file 1 — Supplementary Information and Data [file 41598_2018_34749_MOESM1_ESM.pdf]

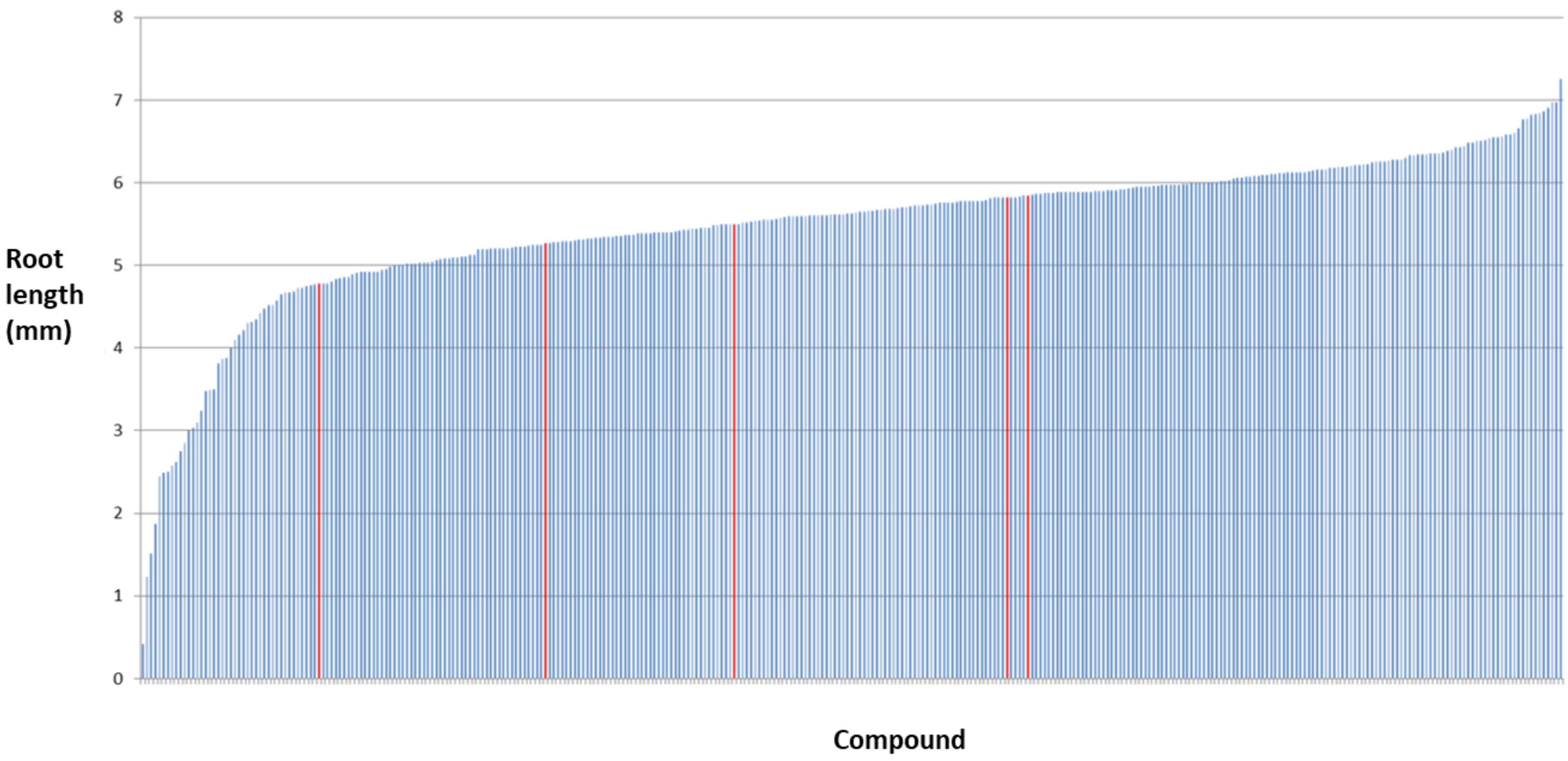

Figure S1. Root length measurements for all library compounds screened against Arabidopsis.
DMSO treated control values are shown in red.

Compound	LogP	# Atoms	Mol Weight	Polar surface area	# O-N	# O-H and N-H	RO5 violation	Molecular volume
1	-2.40	11	163.17	92.94	5	5	0	147.18
2	-0.72	15	219.28	84.15	5	4	0	214.53
3	-2.52	15	221.25	104.38	6	5	0	205.99
4	4.56	28	390.57	61.8	5	2	0	403.01
5	7.59	34	474.73	61.08	5	2	1	503.82
6	-2.04	13	189.21	84.15	5	4	0	107.35
7	2.08	29	411.58	93.38	6	4	0	409.43
8	2.84	26	375.55	93.38	6	4	0	391.53
9	2.77	31	429.56	93.38	6	4	0	416.76
10	2.30	28	397.56	93.38	6	4	0	392.63
11	1.80	27	383.53	93.38	6	4	0	375.83
12	1.38	24	404.30	93.38	6	4	0	329.85
13	0.82	22	319.44	93.38	6	4	0	324.32
14	1.32	23	333.47	93.38	6	4	0	341.13
15	1.83	24	347.50	93.38	6	4	0	357.93

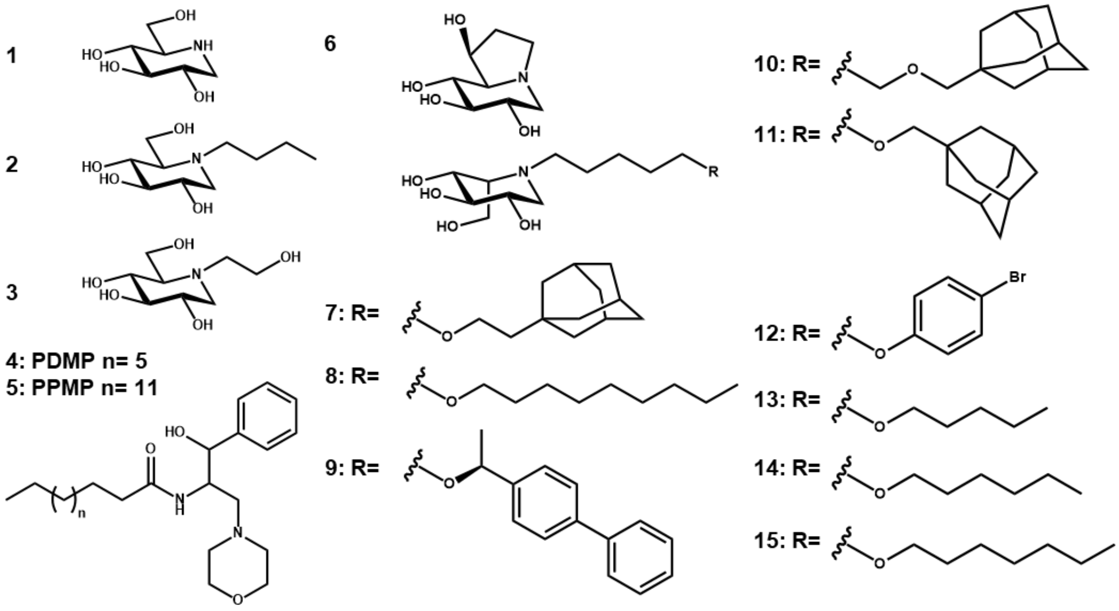

Table S1. Indicators of compound lipophilicity and drug-like criteria.
Data generated using Molinspiration property calculator (http://www.molinspiration.com). The compound numbers refer to those shown in main article figures. LogP is a prediction of the molecules lipophilicity [1]. Polar surface area is a parameter for prediction of drug transport properties determined from the sum of surfaces of polar atoms [2]. RO5 violation relates to the rule-of-5 outlined by Lipinsky et al, molecules violating more than one of these rules may have problems with bioavailability.

1 C.A. Lipinski, F. Lombardo, B.W. Dominy, P.J. Feeney, Experimental and computational approaches to estimate solubility and permeability in drug discovery and development settings. *Adv.Drug.Delivery Rev*. **23**, 4-25 (1997).

2 Ertl, P., Rohde, B., Selzer, P. Fast calculation of molecular polar surface area as a sum of fragment based contributions and its application to the prediction of drug transport properties. *J. Med. Chem*. 2000, **43**: 3714-3717.

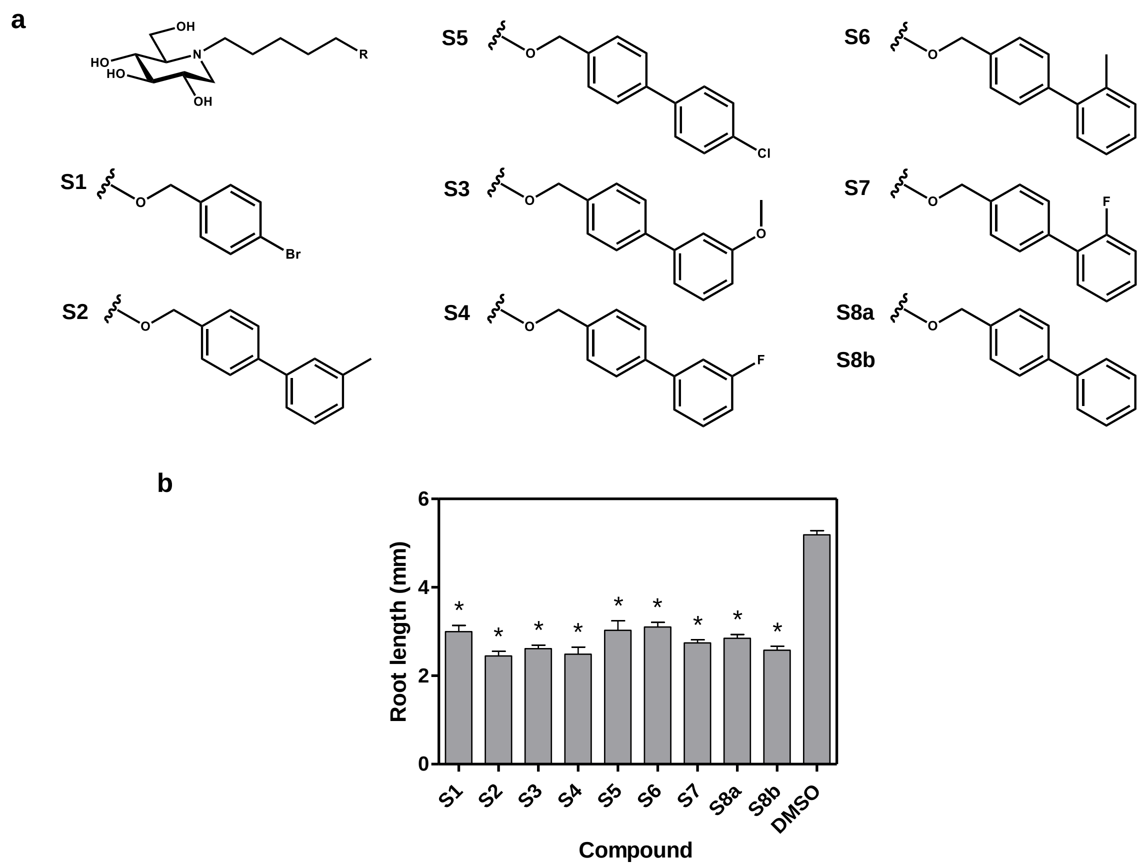

Figure S2. Iminosugar compounds that inhibit Arabidopsis primary root growth when using a threshold between 40-50 % inhibition versus control.

(a) The structures of compounds identified as causing between 40-50 % inhibition of primary root growth in germinating Arabidopsis seedlings. All compounds are *gluco*-configured with aromatic *N*-substituents. (b) Arabidopsis primary root length measurements in the presence of given compounds, seedlings grown for 7 days. Values of root length shown are the mean value for 4-12 seedlings. Error bars represent SEM. Values marked with asterisks are statistically significant from the control (DMSO) values (*t*-test; * $P < 0.05$).

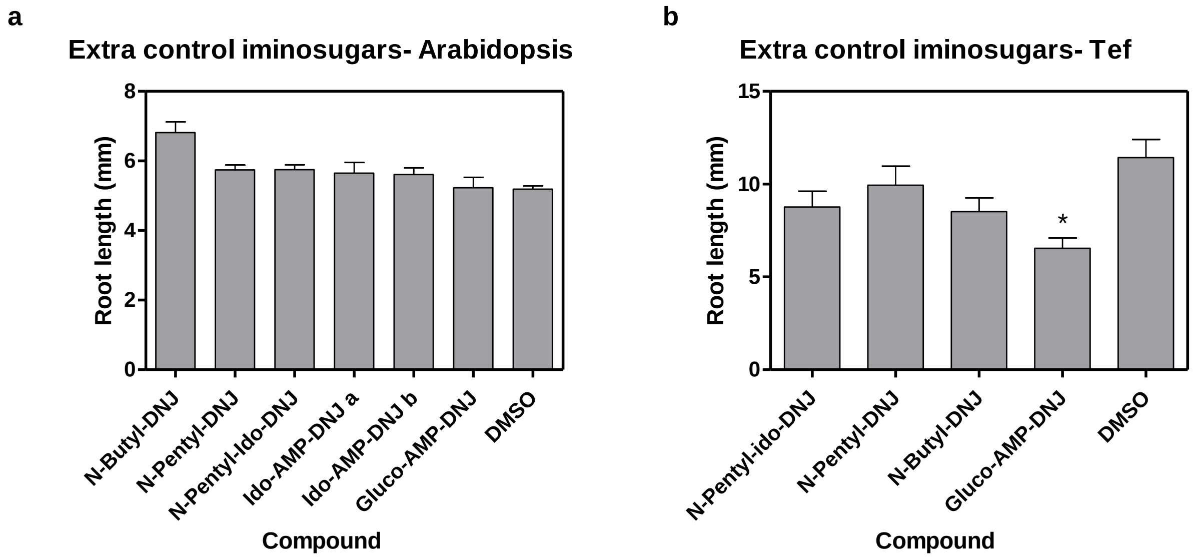

Figure S3. The effect of selected iminosugars on Arabidopsis and Tef primary root growth.
All compounds shown were present in the iminosugar library and tested at a 10 µM concentration. None of the indicated compounds show growth inhibition against Arabidopsis. Tef was unaffected by *N*-pentyl-DNJ, *N*-pentyl-*ido*-DNJ and *N*-butyl-DNJ at 10 µM, however AMP-DNJ showed partial inhibition of root growth. L-*ido*-AMP-DNJ was identified as a strong inhibitor of Tef root growth, data shown in figure 4. Values of root length shown are the mean value for 4-12 seedlings. Error bars represent SEM. Values marked with asterisks are statistically significant from the control (DMSO) values (*t*-test; * P <0.05).

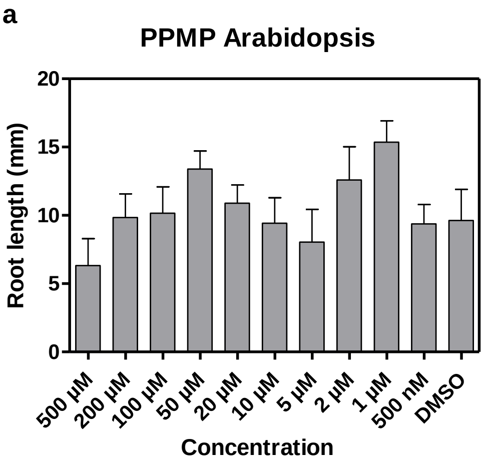

Figure S4. The effect of PPMP on Arabidopsis and Tef primary root growth.
Primary root length measurements in the presence of varying PPMP concentrations (a) Arabidopsis seedlings grown for 7 days. (b) Tef seedlings grown for 3 days. Values of root length shown are the mean value for 4-12 seedlings. Error bars represent SEM. No significant difference between inhibitor treated seedlings and DMSO control was seen (*t*-test; * P <0.05).

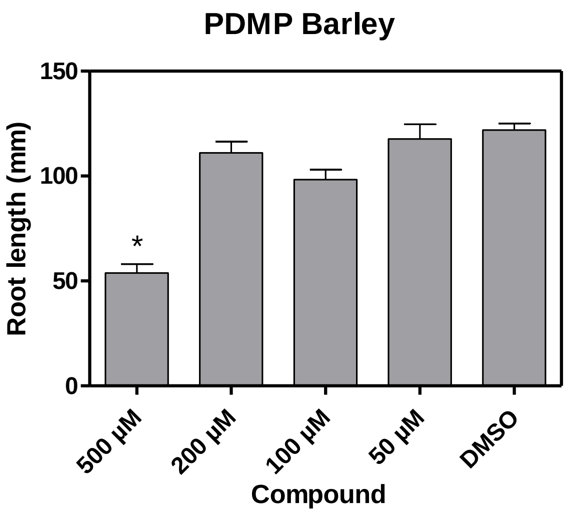

Figure S5. The effect of PDMP on barley primary root growth.
Barley seedling root growth (10 d) was tested using a range of PDMP concentrations. Values of root length shown are the mean value for 10 seedlings. Error bars represent SEM. Values marked with asterisks are statistically significant from the control (DMSO) values (*t*-test; * $P < 0.05$).

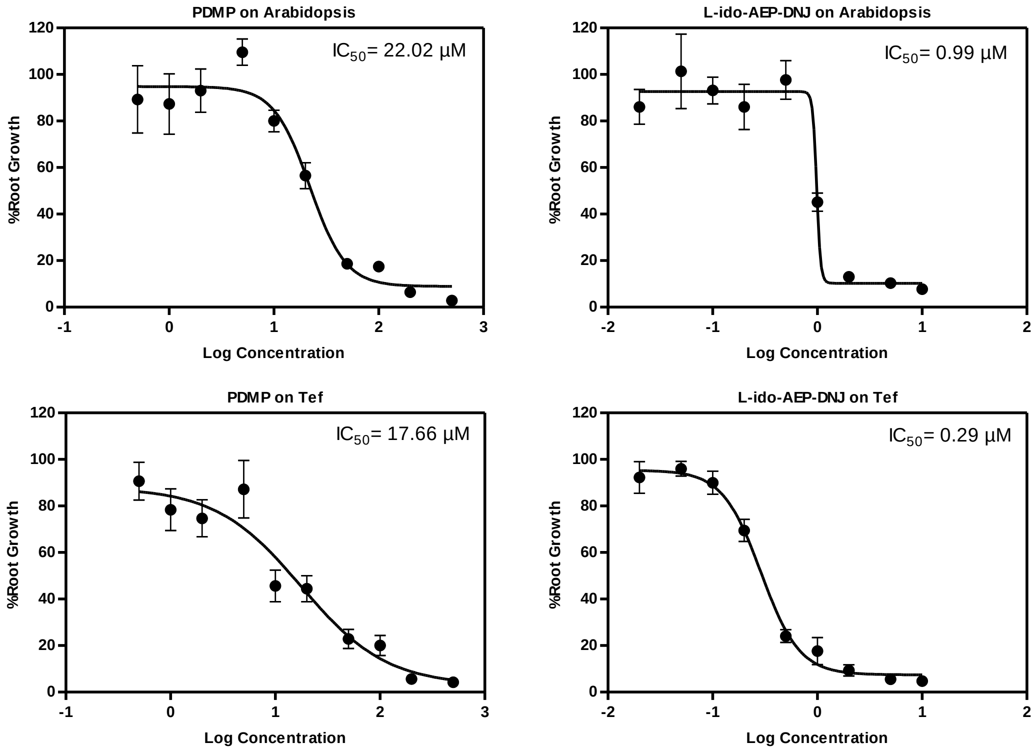

Figure S6. Calculation of IC_{50} values for PDMP and L-*ido*-AEP-DNJ inhibition of Arabidopsis and Tef root growth.

Plotted using root growth data taken from Figure 4.

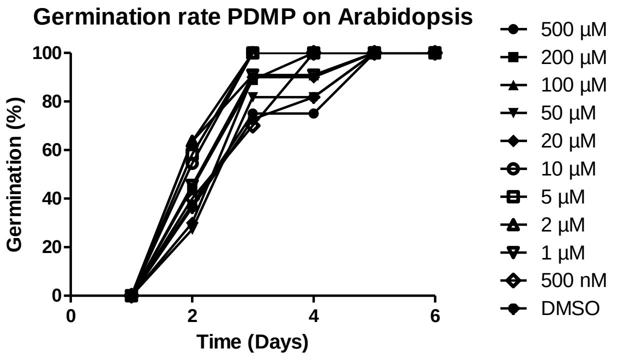

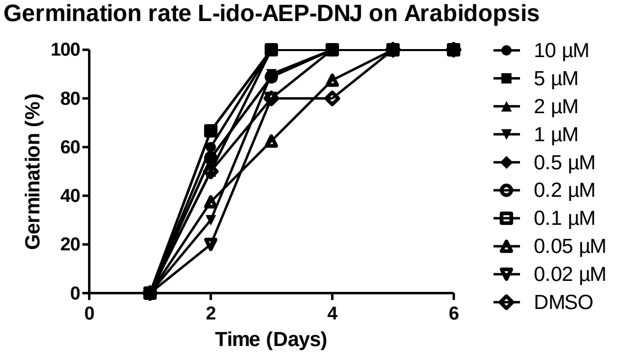

Figure S7. The effect of L-*ido*-AEP-DNJ and PDMP on the germination rate of Arabidopsis.

Arabidopsis seeds were germinated in the presence of inhibitor and radicle emergence was measured at intervals to determine the effect compounds have on germination rate. The compounds were found to have no effect on the rate of germination.

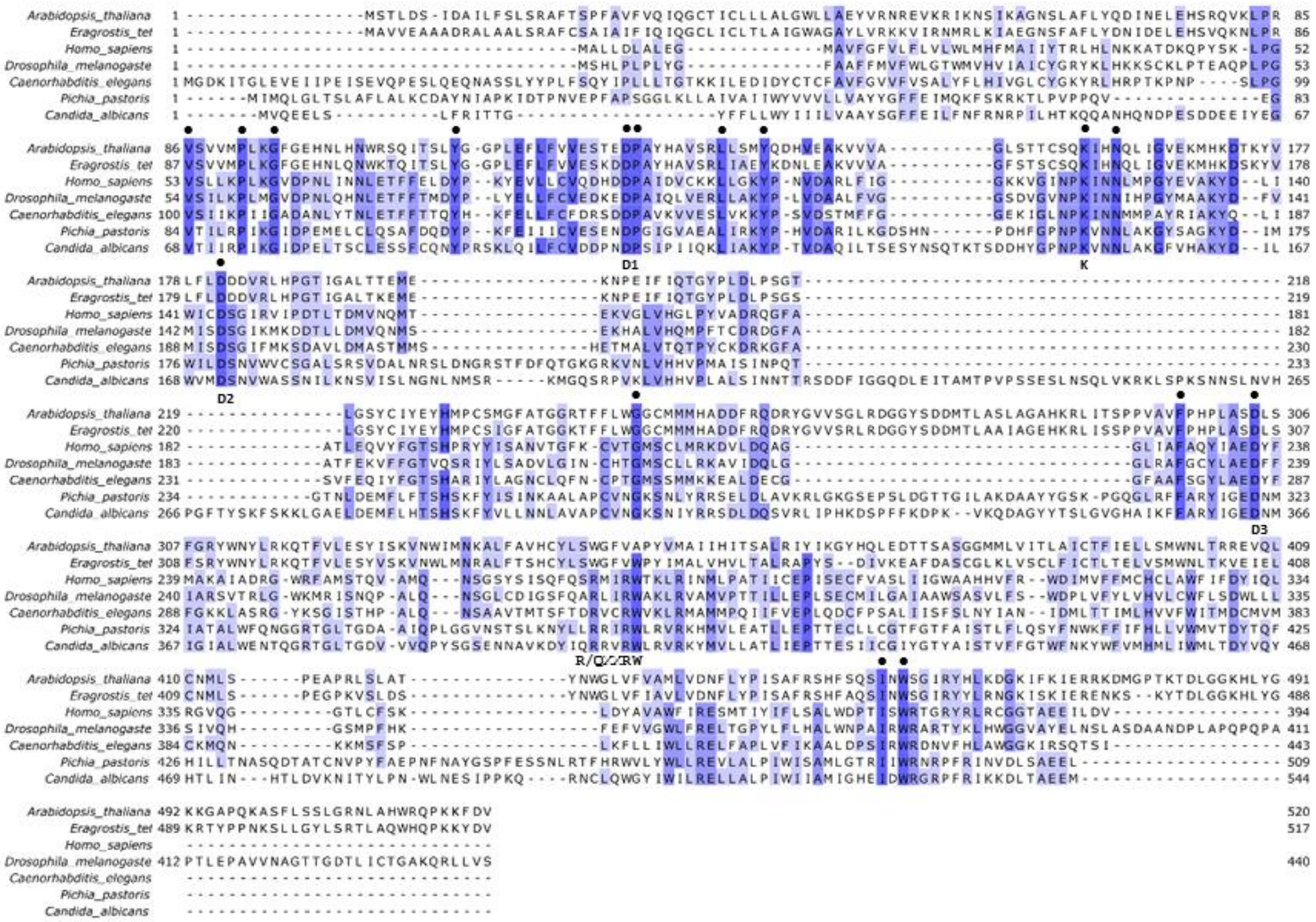

Figure S8. Sequence alignment of eukaryotic glucosylceramide synthase protein sequences.
The overall identity between sequences is between (8-73 %). Conserved residues are shown in blue, darkest tones indicate high similarity. Residues conserved across all sequences are indicated with (•).

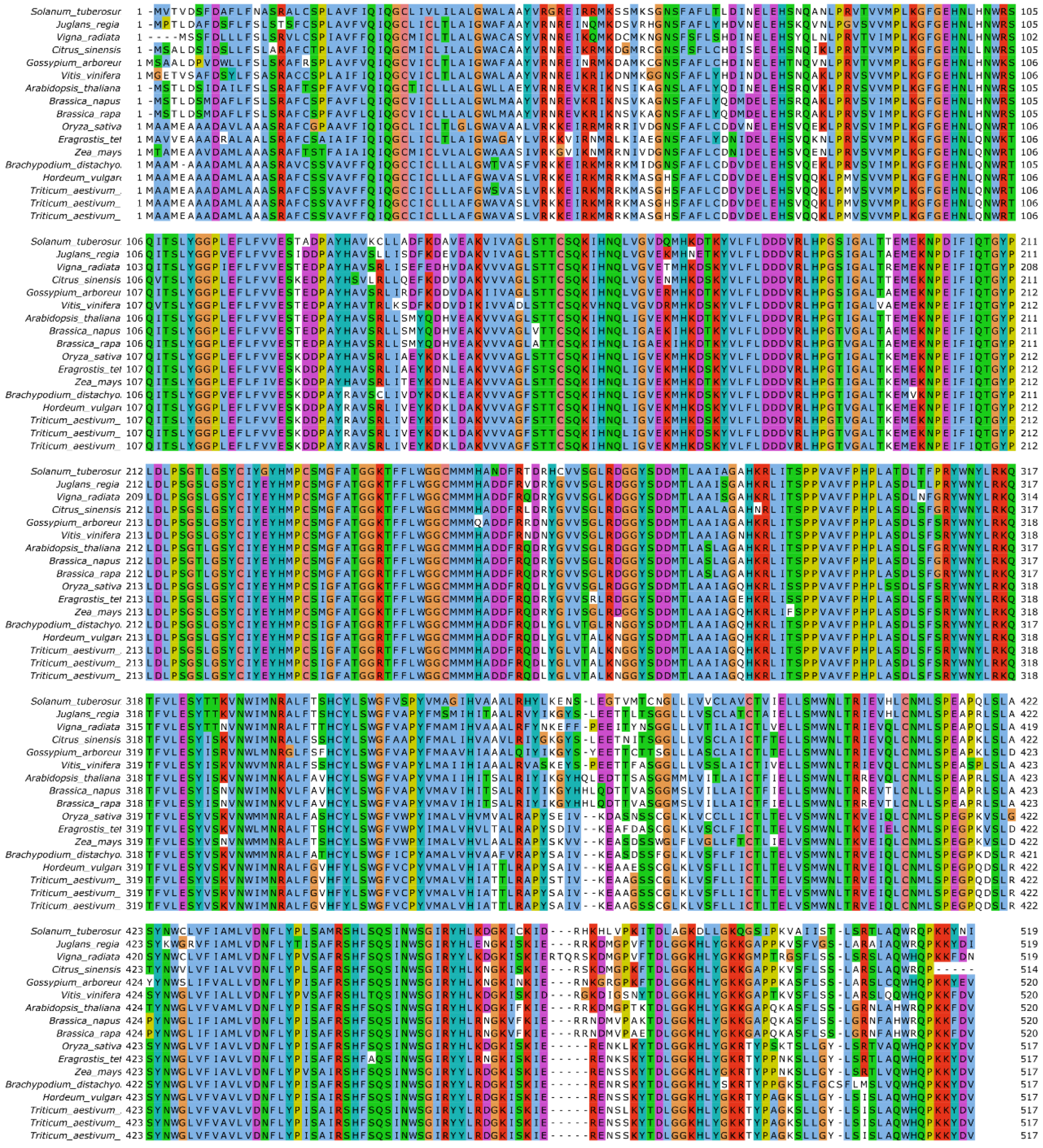

Figure S9. Sequence alignment of plant glucosylceramide synthase protein sequences. Crop and model species were selected to cover a wide variety of plant species. GCS was identified in all plant genomes and transcriptomes analysed. The sequence identity is highly conserved within higher plants, identity between these sequences varies between 69-99 %. Amino acids are represented using the Clustal X colour scheme.

Compound	Parent [m/z]	Daughter [m/z]	Cone Voltage [V]	Collision Energy [V]	Major Product	R_f [min]
C (t18:1; h16:0)	570.5	298.3	34	36	$[LCB-18+H]^+$	10.07
GC (t18:1; h16:0)	732.6	298.3	44	49	$[LCB-18+H]^+$	9.7
C (t18:1; h22:0)	654.6	298.3	34	43	$[LCB-18+H]^+$	12.1
GC (t18:1; h22:0)	816.7	298.3	44	57	$[LCB-18+H]^+$	11.67
C (t18:1; h24:1)	680.7	298.3	50	45	$[LCB-18+H]^+$	12.19
GC (t18:1; h24:1)	842.7	298.3	50	59	$[LCB-18+H]^+$	11.76
C (d18:1; h16:0)	554.5	264.3	40	37	$[LCB-2x18+H]^+$	10.73
GC (d18:1; h16:0)	716.6	264.3	45	53	$[LCB-2x18+H]^+$	10.34
C (d18:1; c12:0)	482.4	264.2	41	20	$[LCB-2x18+H]^+$	9.92
	482.4	464.3	41	10	$[M-18+H]^+$	
	482.4	82	41	44	?	
	482.4	282.2	41	20	$[LCB-18+H]^+$	
GC (d18:1; c12:0)	644.5	264.2	34	28	$[LCB-2x18+H]^+$	9.15
	644.5	626.4	34	12	$[M-18+H]^+$	
	644.5	464.3	34	16	$[M-Glc-18+H]^+$	
	644.5	82	34	64	?	
C (d18:2; h16:0)	552.5	262.2	44	20	$[LCB-2x18+H]^+$	10.47
	552.5	534.4	44	12	$[M-18+H]^+$	
	552.5	94.1	44	34	?	
	552.5	280.2	44	18	$[LCB-18+H]^+$	
GC (d18:2; h16:0)	714.5	262.2	38	30	$[LCB-2x18+H]^+$	10.1
	714.5	696.4	38	8	$[M-18+H]^+$	
	714.5	534.4	38	18	$[M-Glc-18+H]^+$	
	714.5	516.4	38	20	$[M-Glc-2x18+H]^+$	

Table S2: Parameters for MRM detection of diagnostic sphingolipid species and standards used in LC-MS/MS.

Compound abbreviations: Ceramide (C) or glucosylcermides (GC) (LCB; Fatty Acid) where LCB is long-chain base.
